# Do Physical Activity Friendly Neighborhoods Affect Community Members Equally? A Cross-Sectional Study

**DOI:** 10.3390/ijerph15061062

**Published:** 2018-05-24

**Authors:** Nicole E. H. Stappers, Dave H. H. Van Kann, Nanne K. De Vries, Stef P. J. Kremers

**Affiliations:** 1 Department of Health Promotion, NUTRIM School of Nutrition and Translational Research in Metabolism, Maastricht University, P.O. Box 616, 6200 Maastricht, The Netherlands; n.devries@maastrichtuniversity.nl (N.K.D.V.); s.kremers@maastrichtuniversity.nl (S.P.J.K.); 2 School of Sport Studies, Fontys University of Applied Sciences, P.O. Box 347, 5600 AH Eindhoven, The Netherlands; d.vankann@fontys.nl; 3 Department of Health Promotion, CAPHRI Care and Public Health Research Institute, Maastricht University, P.O. Box 616, 6200 Maastricht, The Netherlands

**Keywords:** built environment, physical activity, health inequalities

## Abstract

An activity-friendly environment may increase physical activity (PA) levels and decrease sedentary behavior (SB). This study investigated associations between socio-demographic characteristics, health-related quality of life (HRQoL), perceived environment and objectively measured PA outcomes. Socio-demographic characteristics were assessed using a questionnaire and HRQoL was measured using the EQ-5D. The Neighborhood Environment Walkability Scale (NEWS-A) was used to assess the perceived environment. SB, light PA (LPA) and moderate-to-vigorous PA (MVPA) were measured using the Actigraph GT3X+. Data from 622 Dutch adults were used in multivariate linear regression analyses to investigate associations between NEWS-A and PA outcomes. Analyses were controlled for socio-demographic characteristics and HRQoL. The presence of attractive buildings was associated with less SB (β = −0.086, *p* < 0.01) and more MVPA (β = 0.118, *p* < 0.01). Presence of destinations within walking distance was also positively associated with MVPA (β = 0.106, *p* < 0.01). Less crime was associated with less MVPA (β = 0.092, *p* < 0.05). Interactions between personal and environmental characteristics showed that the absence of PA-hindering characteristics (e.g., heavy traffic) was associated with less SB and more MVPA, but only for residents with problems regarding pain and usual activities. The presence of PA-facilitating characteristics (e.g., aesthetics and destinations) was associated with less SB, more LPA and more MVPA but only for the more advantaged people in society. Results suggest that to reduce health inequalities, it would be more helpful to remove barriers rather than introduce PA facilitating characteristics.

## 1. Introduction

A large body of evidence endorses the effect of socio-economic factors such as educational level, employment and ethnicity on mortality and health [1,2,3,4]. Moreover, individuals with a lower socio-economic status (SES) are at increased risk of adopting unhealthy lifestyle behaviors such as insufficient physical activity (PA) and spending too much time sitting [5]. These unhealthy behaviors increase the risk of obesity and other non-communicable diseases [6,7]. Although insufficient PA levels are seen among all subgroups of society, the likelihood of being obese is higher in disadvantaged individuals [8,9]. It is suggested that socio-economic health inequalities between advantaged and disadvantaged individuals may be related to the built environment in their neighborhood. A multi-country study found that residents of low SES neighborhoods had less favorable perceptions of the environment in their neighborhood, compared to residents of high SES neighborhoods [10], which might lead to lower PA levels and less favorable health-outcomes.

This stresses the need to augment individual-level interventions targeting the increase of PA with interventions at the environmental, policy and societal levels. When designing population-wide interventions, researchers and policymakers should be aware of the differences between “agentic” prevention strategies, in which individuals must use their personal resources, and “structural” strategies, which work through rigorous changes in the total system [11]. Research suggests that treating high-risk individuals with agentic strategies might actually increase health inequalities because disadvantaged individuals can lack the skills needed to change and sustain healthy behaviors [12]. On the other hand, there is increasing evidence to suggest that structural, whole-population approaches generally reduce inequalities [12].

Hence, the built environment is increasingly being used for structural, population-level interventions aiming to increase PA levels and decrease sedentary behavior (SB). Numerous studies explored the associations between the built environment, PA and SB. For example, the presence of bicycling and/or walking infrastructure, the presence of attractive buildings and mixed land-use were found to be associated with increased PA levels [13,14,15,16]. On the other hand, heavy traffic, high crime rates and the presence of physical barriers such as train rails and highways were associated with less PA and more SB [17]. Hereby, built environmental characteristics can be divided into two categories: ones that facilitate PA and ones that hinder PA. The presence of PA-facilitating characteristics and the absence of PA-hindering characteristics potentially lead to more PA and less SB.

Changing built environmental characteristics would thus seem to enable increased PA levels, but adverse effects on PA were reported as well [18,19]. These inconsistencies in the existing literature might be due to varying measuring methods and contexts. An important shortcoming in the current literature is the lack of studies that consider the effect of the built environment on different subgroups in society [18]. It is not clear whether associations between built environment, PA and SB are equitably distributed among advantaged and disadvantaged individuals [18]. As reflected in the hierarchy of walking needs [19], personal characteristics are fundamental when people consider being physically active in their environment. Age, employment, physical functioning and quality of life are some of the factors that contribute to the feasibility of walking. Once individuals experience that it is feasible to walk, then the other layers in the hierarchy become relevant, reflecting characteristics of the built environment.

Therefore, the primary aim of this study was to examine the associations between personal characteristics, perceived environment and objectively measured PA and SB of adults. The secondary aim of this study was to identify possible interactions between perceptions of the built environment and personal characteristics, to assess whether associations between the built environment and PA and SB differ among advantaged and less advantaged groups in society.

## 2. Materials and Methods

### 2.1. Study Design and Sample

Data were collected between September 2016 and July 2017 in two cities in the South-Limburg region of the Netherlands: Maastricht and Heerlen. Ethical approval for the study protocol was granted by the MUMC+ medical ethical committee (METC 16-4-109). The participants provided signed informed consent. Eligible participants (≥18 years, able to walk without walking aids) were reached through social media, posters, flyers, advertisements in local and regional newspapers and personalized mailing. Those who were willing to participate could register via the Internet or by telephone and after registration, participants received an information letter. If they decided to take part, participants were contacted by the researchers by phone or e-mail to plan the distribution of study materials. Study materials were distributed from community centers. After completion of the measurements, researchers collected the study materials from the participants’ homes.

### 2.2. Physical Activity and Sedentary Behavior

The Actigraph GT3X+ accelerometer was used to assess PA levels and SB. Participants were asked to wear the accelerometer during waking hours for seven consecutive days. The Actigraph was worn at the right hip and was only removed during water activities (e.g., bathing, swimming). Raw vector magnitude (VM) data (30 Hz) were downloaded into Actilife version 6.11.7 (Actigraph, Pensacola, FL, USA) and aggregated to 10-s epochs. The Choi algorithm was used to identify wear- and non-wear time [20]. A valid day was defined as at least 10 h of wear time and a valid week consisted of at least 5 valid days, which could include weekends [21]. VM cut-off points were used to distinguish between SB (0–200), LPA (201–2691) and MVPA (>2691) [22,23]. To be able to compare PA levels of our sample with the existing literature, we also calculated SB, LPA and MVPA levels at the vertical axis (VT) using the Freedson 1998 cut-off points [24]. All statistical analyses were performed using the VMpercentage of wear-time spent in SB, LPA or MVPA per day as dependent variable.

### 2.3. Personal Characteristics–Socio-Demographic Characteristics and Health-Related Quality of Life

Participants reported gender, age, household composition, educational level, work status, ethnicity, length and weight. Self-reported length and weight were used to calculate the body mass index (BMI). The EQ-5D questionnaire was used to assess health-related quality of life (HRQoL) in five domains (mobility, daily activities, self-care, pain/complaints and mood), at three levels (no problems, some problems, severe problems) [25]. For all five domains of the EQ-5D a dichotomous variable was created for experiencing no problems (0) or experiencing any/severe problems (1).

### 2.4. Environmental Characteristics

The perceived environment was measured using a variety of subscales of the abbreviated version of the Neighborhood Environment Walkability Scale (NEWS-A) [26]. The NEWS-A has shown to be a valid and reliable measure for neighborhood walkability [27]. Translated versions of the NEWS-A have been used in several studies in the Netherlands and Belgium [28,29]. The following subscales were included in the questionnaire: Access to facilities, aesthetics, infrastructure and safety for walking, traffic hazards, crime, lack of parking spaces, hilliness, and physical barriers. Although all items say something about the activity-friendliness of a neighborhood, we distinguished between “PA facilitating characteristics” or “PA hindering characteristics”. All NEWS-A items were scored on a 4-point scale and if necessary, items were recoded in order to create scales in which higher scores reflected a more activity-friendly environment. Scores ranged from 1 (not activity friendly environment) to 4 (very activity friendly environment).

### 2.5. Statistical Analyses

Statistical analyses were conducted using SPSS version 23 (IBM Corp., Armonk, NY, USA). Descriptive statistics were used to describe sample characteristics and mean values and standard deviations of socio-demographic characteristics, HRQoL, perceived environment, perceived health, PA and SB. Also, all variables were checked on multicollinearity by assessing the correlation matrix and by calculating the variance inflation factor in SPSS. Both did not indicate problems regarding multicollinearity.

Associations between personal characteristics, environmental characteristic, and PA outcomes were explored using multivariate linear regression analyses. To assess the main effects of environmental characteristics on SB, LPA and MVPA, we used a hierarchal regression method with two blocks. The first block contained socio-demographic characteristics and the five domains of HRQoL. The second block contained the items of the NEWS-A. The backward deletion method was used for each block to exclude the least significant variables until all remaining variables were statistically significant (*p* < 0.05). Model 1 contains significant personal characteristics and model 2 contains significant personal and environmental characteristics. All analyses were performed using VM PA outcomes.

Additional moderation analyses were performed to explore possible interactions between personal and environmental characteristics. First, interaction terms were calculated for each possible interaction between socio-demographic characteristics and HRQoL, and environmental characteristics. All possible interaction terms were individually added to model 2, to identify interactions that contributed significantly to the model, independently of other interaction terms. Next, for each outcome measure (SB, LPA and MVPA), model 2 was augmented by a third block containing the detected significant interaction terms. The backward deletion method was used to exclude the least significant variables, with exception of main effects of significant interaction terms.

To interpret detected interactions, we performed stratified analyses on the significant interactions and visualized these findings.

## 3. Results

### 3.1. Participants’ Characteristics

In total, 758 participants were included in this study. Thirty-seven participants (5%) were excluded because of missing questionnaire data and 99 participants (13%) did not provide a valid PA measurement over at least 5 valid days, leaving 622 participants in the final sample. Table 1 presents the characteristics of the participants. The mean BMI-index was 24.9 ± 4.2 kg/m^2^. Although 99% (*n* = 616) of the participants did not experience any problems regarding self-care, about 12% (*n* = 75) experienced problems regarding mobility, 10% (*n* = 62) reported problems with usual activities and 10% (*n* = 62) reported experiencing moderate or extreme problems regarding anxiety/depression. Also, 31% (*n* = 193) reported moderate or extreme problems regarding pain/discomfort.

### 3.2. Physical Activity Levels

The mean weartime of the accelerometers was about 14.5 h per day. Average percentage of the day and mean time (minutes/day) spent in SB, LPA, and MVPA calculated using VM and VT counts are presented in Table 2.

### 3.3. Neighborhood Environment Walkability Scale

Table 3 shows the mean scores on the items of the NEWS-A questionnaire. Scores on all items ranged from 1.0 to 4.0, with a higher mean score reflecting a higher perceived activity-friendly environment. Means ranged from 2.0 for the presence of a grass/dirt strip that separates streets and sidewalks, the amount of traffic, and the speed of traffic, to 3.5 for the presence of stores within walking distance.

### 3.4. Associations between SB, LPA, MVPA and Personal and Environmental Characteristics

Table 4 shows the associations between SB, LPA, MVPA, personal (socio-demographic variables and HRQoL) and environmental characteristics.

Participants with a higher BMI and participants experiencing any/severe problems with self-care showed more SB compared to participants with a lower BMI and participants without problems regarding self-care. Women, lower educated participants, and participants with children in their household showed less SB compared to men, higher educated participants and participants without children in their household. When controlling for those significant personal characteristics, the presence of attractive buildings in the resident’s neighborhood was associated with less SB.

Women, older participants, lower educated participants and participants with children in their household showed more LPA compared to men, younger participants and participants without children in their household. LPA was negatively associated with BMI and problems with self-care, meaning that participants with a higher BMI and any/severe problems regarding self-care showed less LPA compared to participants with a higher BMI and participants that experience any/severe problems regarding self-care. After controlling for significant personal characteristics, no environmental characteristics were associated with LPA.

Being lower educated and experiencing pain/discomfort was associated with more MVPA. Older participants, participants with a higher BMI, experiencing any/severe problems regarding self-care and participants experiencing any/severe problems regarding usual activities were associated with less MVPA. When adding environmental variables to the model, the presence of places to go within walking distance and the presence of attractive buildings in the neighborhood were positively associated with MVPA, while a higher score on perceived crime, indicating less crime, was negatively associated with MVPA.

### 3.5. Interactions between Personal and Environmental Characteristics

**Figure 1:**
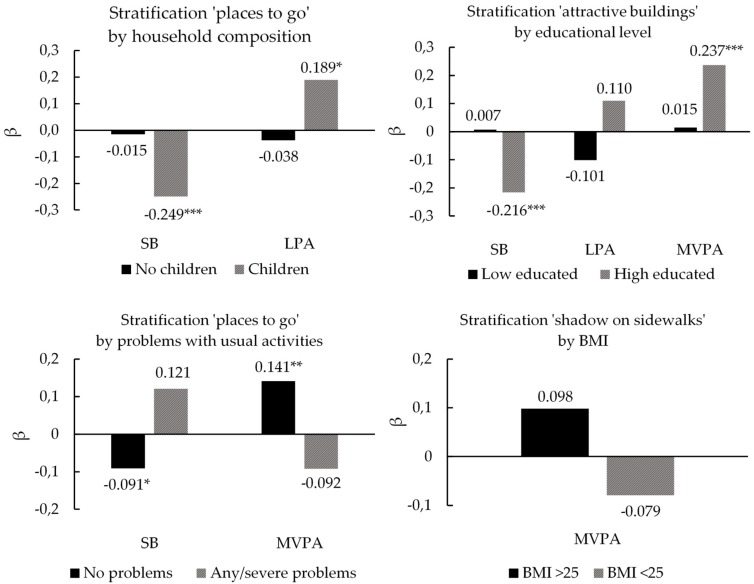
Interactions between personal characteristics (socio-demographic and HRQoL) and PA-facilitating built environmental characteristics. * = *p* < 0.05; ** = *p* < 0.01; *** = *p* < 0.001.

For the PA-facilitating characteristics, we found the following significant interactions: household composition x places to go (β = −0.517, *p* = 0.003), educational level x attractive buildings (β = −0.496, *p* < 0.001) and usual activities x places to go (β = 0.331, *p* = 0.029). We did not detect main associations for the presence of shadows on sidewalks (model 2), but we detected an interaction between BMI and shadow on sidewalks (β = −0.842, *p* = 0.002). The presence of places to go within walking distance was associated with decreased time spent in SB and increased time spent in LPA for participants living in a household with children (β = −0.249, *p* < 0.01 and β = 0.189, *p* < 0.05, respectively) and less time spent in SB and more time spent in MVPA for participants experiencing no problems regarding usual activities (β = −0.091, *p* < 0.05 and β = 0.141, *p* < 0.01, respectively). The presence of attractive buildings was significantly associated with less SB and more MVPA for higher educated residents (β = −0.216, *p* < 0.001 and β = 0.237, *p* < 0.001, respectively), but no significant associations were found for LPA nor for lower educated participants. Stratification analyses of significant interactions between personal and PA facilitating environmental characteristics are illustrated in Figure 1.

**Figure 2:**
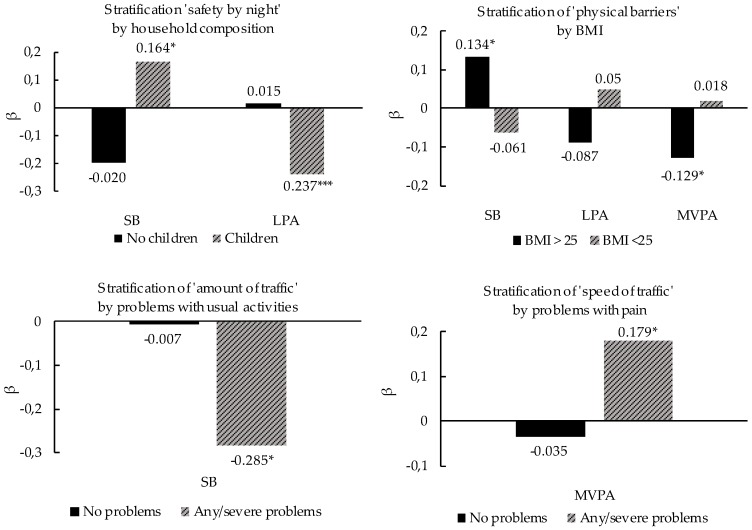
Interactions between personal characteristics (socio-demographic and HRQoL) and PA-hindering built environmental characteristics. * = *p* < 0.05; *** = *p* < 0.001.

For PA-hindering characteristics, we found four significant interactions as well. A better perceived safety at night was associated with more SB and less LPA for households with children (β = 0.164, *p* < 0.05 and β = −0.237, *p* < 0.001 respectively). Fewer perceived physical barriers were significantly associated with more SB and less MVPA for participants with a BMI higher than 25.0 (β = 0.134, *p* < 0.05 and β = −0.129, *p* < 0.05, respectively). For households without children and participants with a BMI lower than 25.0, these associations were not significant. For residents experiencing any/severe problems with usual activities, less perceived traffic was associated with less SB (β = −0.285, *p* < 0.05). For participants experiencing any/severe pain, less perceived high speed traffic was associated more MVPA (β = 0.179, *p* < 0.05). There were no significant associations for participants experiencing no problems. Stratification analyses of significant interactions between personal and PA hindering environmental characteristics are illustrated in Figure 2.

## 4. Discussion

This study explored the associations between socio-demographic characteristics, health-related quality of life (HRQoL), perceived environmental characteristics and PA outcomes. The presence of attractive buildings and the presence of places to go in one’s neighborhood were associated with less SB and more MVPA in the total sample. However, our findings suggest that for less advantaged residents in society, it would be more helpful to remove barriers such as heavy traffic rather than introduce PA-facilitating characteristics such as the presence of destinations in the neighborhood and improved aesthetics.

Consistent with other studies, we found that having a higher BMI and experiencing any/severe problems regarding self-care and usual activities were associated with more SB and less LPA and MVPA [30,31,32]. Living with children was associated with less SB and more LPA compared to living without children, which was also shown in a recent systematic review [33]. In contrast to the majority of the existing literature, we found that women engaged in less SB and more LPA [9]. This exchange between SB and LPA might indicate that women substitute sitting by activities such as cleaning and taking care of children, which are all considered to be light physical activities [34]. We also found that experiencing any/severe pain or discomfort was associated with more MVPA compared to those who did not experience any pain/discomfort. An explanation for this unexpected finding could be that participants with complaints of pain might be engaged in physical therapy, leading to more exercise-related MVPA during the week. A U-shaped association between chronic pain and PA has been suggested [35], in which small amounts and very large amounts of PA are associated with chronic pain.

LPA was only associated with personal characteristics and not with environmental ones. This might be explained by the nature of light physical activities, which are often indoor activities such as household chores or caring for children [34]. These indoor activities are possibly less affected by perceptions of the environment compared to outdoor activities.

With respect to environmental characteristics, our study showed that the presence of attractive buildings in the neighborhood was associated with less SB and more MVPA, which is supported by previous literature [14,36,37]. Remarkably, another European study found that living in a neighborhood with a higher score for perceived aesthetics was associated with more total self-reported sedentary behavior [38]. These contrasting findings in a fairly comparable sample and context could be due to differences in measuring methods for SB or the difference between neighborhood- and individual-level information on aesthetics.

The presence of places to go within walking distance was associated with more MVPA, which was also supported by previous studies [14,37]. We unexpectedly found that a higher score for crime, indicating less perceived crime in the neighborhood, was associated with less MVPA, which contradicts the majority of the literature findings [17]. A possible explanation might be that the more active residents are also more aware of the crime in their neighborhood compared to their sedentary counterparts. It could also reflect a lack of alternatives to walking for those living in an area with more crime [39]. It might be helpful to add objective crime rates to investigate this association in more detail [17,32].

The majority of the NEWS-A variables were not associated with SB, LPA or MVPA, after controlling for personal characteristics (socio-economic demographics and HRQoL). This might be due to the difference in the specificity of the PA measurements and the NEWS-A questionnaire. The NEWS-A assessed the perceived activity-friendliness of the participants’ neighborhood, but PA measurements were not restricted to a specific area. Context-specific PA measurements might help to clarify the relation between perceived neighborhood environment and PA outcomes, based on the actual exposure to the neighborhood environment.

The presence of PA-facilitating characteristics such as attractive buildings and places to go within walking distance were associated with less SB and more MVPA in the total sample. However, moderation analyses showed that these associations were only significant for higher educated individuals and individuals without problems regarding usual activities. For the more vulnerable individuals in society, we did not find these significant associations and stratified analyses even showed an opposing trend. Explorative analyses of our data showed that even in some area-level low-SES neighborhoods the higher educated participants scored higher on the presence of attractive buildings, compared to their lower educated counterparts.

For the more vulnerable individuals experiencing problems with usual activities and pain, we found that fewer PA-hindering characteristics, such as heavy and speeding traffic, resulted in less SB and more MVPA. There was no significant association for residents without problems. This indicates that traffic is a PA-hindering factor for people who experience problems, while people without problems can overcome these barriers in daily life. This contrasts somewhat with the findings of Carlson et al. (2014), who reported that PA levels of the more advantaged individuals may be impacted more by neighborhood safety compared to the less advantaged individuals [40]. However, Carlson and colleagues used educational level, ethnicity and income for interaction terms, rather than quality of life.

Our findings do not support the statement of Mertens et al. (2017), that built environmental interventions could be positive for everyone or at least do not disadvantage subgroups [41]. Indeed, the presence of attractive buildings and the presence of destinations within walking distance can improve PA levels for the total population, but from a socio-economic health inequality perspective, the opposite trend between PA-facilitating characteristics and PA for the less advantaged residents indicates the need for specific research attention. Our results suggest that structural interventions implementing PA-facilitating features could increase socio-economic health inequalities because they may lead to increased PA levels of the more advantaged individuals only. Longitudinal, experimental studies are necessary to confirm these results, and they should be taken into account when designing interventions targeting the reduction of socio-economic health inequalities.

Two interactions showed associations that were difficult to explain. The absence of physical barriers (railroads, rivers, highways), was associated with more SB and less MVPA in overweight residents, while an opposite but non-significant trend was detected for people with a normal weight. For residents in a household with children, more safety at night was associated with more SB and less MVPA, compared to residents in a household without children. The use of context-specific analyses in which the location of PA behavior is included, might be helpful to examine whether there is a logical pattern explaining these unexpected findings, our whether these findings are more likely to be type I errors.

The strength of this study is the objective measurement of PA and SB outcomes. In contrast to the majority of the studies, this study included HRQoL as a possible moderator of PA and paid attention to health inequalities that might be influenced by the environment.

An important limitation of this study is its cross-sectional design, which makes it impossible to detect causal relationships. The percentage of non-western residents who participated was too low to control for ethnicity in the models, which affects generalizability of the results. The lack of variation on the NEWS-A item “presence of sidewalks” was very low: 96% (*n* = 597) of the participants agreed or strongly agreed with the statement that sidewalks were present in their neighborhood. Therefore, we decided to exclude this variable from our analyses. Moreover, the sample was fairly high educated (53%) and retired adults might be overrepresented in the sample. Compared to other studies [42,43], PA levels were generally high. This indicates that the recruitment strategies led to increased self-selection of active adults into the study. We found differences in MVPA levels between VM and VT calculations, though the relative difference between the two methods was comparable with other studies [43,44]. This emphasizes a limitation of the use of accelerometry in PA research. There are various data-processing protocols and different cut-off values that affect the estimation of the actual energy expenditure. Also, cut-off points are developed in relatively controlled research settings, which might induce inaccuracies when using them in a free-living environment [45].

Lastly, the large number of possible interactions between personal and environmental characteristics that were tested increased the chance of type I errors, i.e., detecting interactions that are not present. To reduce this risk, the suggested significance level for interaction terms of α < 0.10 [46] was reduced to α < 0.05. We found more significant interaction terms than expected based on coincidence.

## 5. Conclusions

More PA-supportive environments such as the presence of places to go within walking distance and the presence of attractive buildings can lead to a decrease in SB and an increase in MVPA, but might not affect LPA. However, the potential of the built environment to affect PA outcomes may differ for advantaged and disadvantaged individuals in society. To design structural built environmental interventions that reduce socio-economic health inequalities, more context-specific research and (natural) experiments are needed. These studies can help to investigate causal relationships between the socio-demographic characteristics, health related quality of life, environmental characteristics and PA outcomes.

## Figures and Tables

**Table 1:** Participants’ characteristics.

**Socio-Demographic Characteristics (*n* = 622)**	**%/Mean (±SD)**
Gender (% Males)	46%
Age (years)	57.3 (15.6)
Educational level (% Higher educated) *	54%
Work status (% Employed)	48%
Ethnicity (% Western)	98%
Household composition	
without children	76%
with children	24%
Body Mass Index (kg/m^2^)	24.9 (4.2)
**Health-related quality of life (*n* = 622)**	
Mobility	
no problems	88%
moderate problems	12%
extreme problems	0%
Self-care	
no problems	99%
moderate problems	1%
extreme problems	<1%
Usual activities	
no problems	90%
moderate problems	10%
extreme problems	<1%
Pain/discomfort	
no problems	69%
moderate problems	30%
extreme problems	1%
Anxiety/depression	
no problems	90%
moderate problems	9%
extreme problems	1%

* Higher educated participants have a higher professional education or university degree.

**Table 2:** Participants’ physical activity levels based on vector magntiude (VM) and vertical axis (VT) calculations.

Physical Activity Levels (*n* = 622)	% (±SD)	Mean minutes/Day (±SD)
Wear time		868.5 (196.0)
Vector magnitude		
% Sedentary behavior	65.4 (7.8)	567.5 (98.1)
% Light physical activity	26.1 (6.2)	227.5 (64.0)
% Moderate-to-vigorous physical activity	8.4 (3.7)	73.4 (34.0)
Vertical axis		
% Sedentary behavior	74.7 (6.2)	647.7 (99.7)
% Light physical activity	19.9 (5.3)	173.4 (53.5)
% Moderate-to-vigorous physical activity	5.5 (4.9)	47.4 (25.7)

**Table 3:** Mean scores (±SD) per item of NEWS questionnaire.

Scale	Variable	Mean * (±SD)
PA facilitating characteristics		
Access to facilities	Stores within easy walking distance	3.5 (0.7)
	Many places within walking distance	3.2 (0.7)
	Easy to walk to transit stop (bus, train)	3.4 (0.7)
Infrastructure and safety for walking	Cars dividing sidewalk and traffic	2.9 (0.6)
	Grass/dirt dividing sidewalk and traffic	2.0 (0.7)
	Street lights	3.1 (0.5)
	Walkers and bikers easily seen	3.0 (0.5)
	Crosswalks and pedestrian signals	2.6 (0.8)
Aesthetics	Trees	3.1 (0.8)
	Many interesting things to look at	2.5 (0.7)
	Many attractive natural sights	2.6 (0.8)
	Attractive buildings/homes	2.5 (0.8)
PA hindering characteristics		
Traffic hazards	Amount of traffic	2.0 (0.7)
	Speed of traffic	2.0 (0.7)
	Drivers exceed posted limits	2.7 (0.8)
Crime	High crime rate	2.9 (0.6)
	Crime rate makes it unsafe during the day	3.3 (0.6)
	Crime rate makes it unsafe at night	3.0 (0.7)
Lack of parking	Parking is difficult	2.1 (0.8)
Hilliness	Hilliness	3.4 (0.6)
Physical barriers	Physical barriers	3.4 (0.7)

* All items ranged from 1 to 4, in which a higher score is considered to be more PA support.

**Table 4:** Multivariate linear regression models for SB, LPA and MVPA.

Variables	SB		LPA		MVPA	
	Model 1	Model 2	Model 1	Model 2	Model 1	Model 2
	β	β	β	β	β	β
**Personal characteristics**						
Gender-female (REF = male)	−0.156 ***	−0.154 ***	0.199 ***	0.199 ***		
Age			0.158 ***	0.158 ***	−0.132 **	−0.141 **
Body Mass Index	0.182 ***	0.177 ***	−0.179 ***	−0.179 ***	−0.127 **	−0.115 **
Educational level-low (REF = high)	−0.182 ***	−0.195 ***	0.139 ***	0.139 ***	0.122 **	0.120 **
Household composition-with child (ren) (REF = no children)	−0.139 ***	−0.130 **	0.184 ***	0.184 ***		
Self-care-any problems (REF = no problems)	0.150 ***	0.150 ***	−0.113 **	−0.113 **	−0.104 **	−0.099 *
Usual activities-any problems (REF = no problems)					−0.158 **	−0.160 ***
Pain-any problems (REF = no problems)					0.100 *	0.102 *
**Explained variance (R^2^)**	0.143		0.150		0.078	
**Environmental characteristics**						
Places to go within walking distance (higher score, more places to go)						0.106 **
Attractive buildings (higher score, more attractive buildings)		−0.086 **				0.118 **
Crime (higher score, less crime)						−0.092 *
**Explained variance (R^2^)**		0.150		0.150		0.113

REF = reference category; * = *p* < 0.05; ** = *p* < 0.01; *** = *p* < 0.001.
